# Editorial: Stem cells in pancreatic cancer

**DOI:** 10.3389/fonc.2023.1248807

**Published:** 2023-07-10

**Authors:** Enza Lonardo

**Affiliations:** Gastrointestinal Cancer Laboratory, Institute of Genetics and Biophysics, Consiglio Nazionale delle Ricerche (CNR), Naples, Italy

**Keywords:** pancreatic cancer, tumor organoids, extracellular matrix (ECM), exosome, EMT

Pancreatic cancer (PC) is a devastating disease with limited treatment options and high mortality rates. Enhancing therapeutic strategies and accurately predicting treatment response are crucial for improving patient outcomes.

This Research Topic focuses on five studies that explore innovative approaches using pancreatic tumor organoids (PTOs), investigate prognostic biomarkers like damage-specific DNA binding protein 2 (DDB2), examine the COL10A1-DDR2 axis in PC progression, unravel exosome-mediated chemoresistance transmission, and explore the role of small nucleolar RNA host gene 1 (SNHG1) in PC progression. These studies provide valuable insights into the potential of organoid models and molecular biomarkers for advancing PC therapies.

PTOs are 3D models derived from patient-derived tumor tissues that closely mimic the characteristics of pancreatic tumors. These organoids offer several advantages over traditional 2D cell culture models, providing a better representation of tumor architecture, cellular heterogeneity, and tumor-stromal interactions. PTOs have demonstrated great potential in evaluating tumor response to radiation and drug treatments, allowing for the development of personalized therapeutic strategies. Moreover, they enable the exploration of tumor biology, including cancer stem cells and tumor heterogeneity. By serving as a more accurate and relevant platform, PTOs represent a significant advancement in pancreatic cancer research, offering insights into tumor behavior and treatment response that can potentially improve patient outcomes. In the study conducted by Shukla et al., the focus was on utilizing mouse-derived pancreatic tumor organoids to assess their responses to radiation and drug treatments. The findings of their investigation demonstrated that PTOs closely mimic the fibrotic microenvironment and molecular responses observed in *in vivo* tumors. This indicates that PTOs have the potential to be a valuable tool for predicting tumor sensitivity and response to radiation and combined chemo-radiotherapy.

Damage-specific DNA binding protein 2 (DDB2) is a critical protein involved in DNA repair and tumor suppression. In their study, Dardare et al. specifically investigated the role of DDB2 in PC. Their findings revealed that reduced expression of DDB2 in PC tissues was associated with shorter disease-free survival in patients. To gain further insights into the functional aspects of DDB2, the researchers employed CRISPR-modified PC cell models. The results demonstrated that overexpression of DDB2 inhibited epithelial-to-mesenchymal transition (EMT), migration, invasion, and enhanced chemosensitivity in PC cells. These significant findings highlight the potential of DDB2 as both a therapeutic target and a prognostic biomarker in PC. The use of CRISPR-modified cell models to understand the role of DDB2 in PC opens up opportunities for the development of novel treatment strategies and the improvement of patient outcomes.

Collagen is a major component of the extracellular matrix (ECM) and provides structural support to tissues. In PC, aberrant collagen deposition and remodeling occur, leading to changes in the tumor microenvironment. Collagen accumulation in pancreatic tumors can create a dense and fibrotic stroma, known as desmoplasia. This desmoplastic reaction promotes tumor growth, angiogenesis, and immune cell infiltration. Additionally, the altered ECM composition affects tumor cell behavior, including proliferation, migration, invasion, and resistance to therapy. Collagen-related proteins, such as COL10A1, have been implicated in PC progression and poor prognosis. In the study of Wen et al., the researchers focused on investigating the COL10A1-DDR2 axis in PC progression. They found that increased COL10A1 expression in PC cells and tissues correlated with a poor prognosis. Through functional experiments, the study demonstrated that COL10A1 overexpression promoted PC cell proliferation and migration. Furthermore, the study revealed that COL10A1 positively regulated DDR2 expression and activation, leading to the activation of the MEK/ERK pathway and EMT, both of which are involved in PC progression. Targeting the COL10A1-DDR2 axis and downstream MEK/ERK signaling pathways could potentially offer novel therapeutic strategies for PC treatment.

Exosomes are small vesicles released by cells that contain various molecules, including proteins, nucleic acids, and lipids, and play a role in intercellular communication. MMP14, also known as matrix metalloproteinase 14 is an enzyme belonging to the matrix metalloproteinase family. MMPs are a group of enzymes involved in the degradation and remodeling of the ECM. MMP14 specifically plays a significant role in various physiological and pathological processes, including cancer. Li et al. conducted a study focusing on the role of exosome-transferred MMP14 in gemcitabine resistance in PC cells. They found that MMP14 carried by exosomes enhanced drug resistance, cancer stemness, sphere-formation, and migration abilities in recipient PC cells. This suggests that the transfer of MMP14 through exosomes contributes to the development of resistance against gemcitabine, a commonly used chemotherapy drug for PC. Targeting MMP14 in exosomes may offer a potential therapeutic approach to overcome gemcitabine resistance in PC. Additionally, exosome-transferred MMP14 could serve as a biomarker to predict the response of PC patients to gemcitabine therapy, allowing for personalized treatment strategies.

SNHG1 is a small nucleolar RNA host gene that plays a role in the progression of various cancers, including PC. It is located on human chromosome 11 and is known to be dysregulated in several malignancies. SNHG1 has been found to be upregulated in various cancer cells and tissues, suggesting its involvement in disease development and progression. Chen et al. conducted a study to investigate the involvement of SNHG1 in PC progression. They found that downregulation of SNHG1 inhibited cell proliferation and induced apoptosis in PC cells. The study also revealed that SNHG1 influenced the migration and invasion of PC cells by affecting the expression of EMT-related proteins. Moreover, the study demonstrated that SNHG1 acted as a competitive endogenous RNA, positively regulating the expression of fibroblast growth factor receptor 1 (FGFR1) by sponging microRNA-497 (miR-497). Downregulation of miR-497 was associated with poor prognosis in cancer patients. SNHG1 upregulated FGFR1 expression by sequestering miR-497, promoting PC progression. These findings suggest that SNHG1 could serve as a potential therapeutic target for PC treatment.

In conclusion, the studies discussed in this editorial provide valuable insights into the role of organoid models and molecular biomarkers in advancing pancreatic cancer research. These findings contribute to the development of novel therapeutic strategies and personalized approaches for the treatment of PC. Continued research in this field will further enhance our understanding of pancreatic cancer biology and improve patient outcomes.

## Author contributions

The author confirms being the sole contributor of this work and has approved it for publication.

